# Dialectical behaviour therapy in the treatment of borderline personality disorder: a commentary

**DOI:** 10.7748/mhp.2025.e1729

**Published:** 2025-03-11

**Authors:** K Gomez Ujhelyi, E Rushton, J Harrison, JE Edward

**Affiliations:** University of Liverpool; Lancashire and South Cumbria NHS Foundation Trust; University of Lancashire

## Abstract

Dialectical Behaviour Therapy (DBT) was designed specifically to treat deliberate self-harm (with or without suicide intent) in women with borderline personality disorder (BPD). It has received the most research attention compared to other psychological approaches in this area. The aim of this commentary is to critically appraise the evidence provided by a systematic review on the effects of DBT on self-harm, suicidal ideation, anger, and depression in individuals with BPD. The methods used was Rapid Conversion of Evidence Summaries, a collaborative and educational approach between academics and health and care professionals to understand and summarise research evidence and its implications to practice and policy. As such, the work involved the critical appraisal of a systematic review relevant for the mental health nurse’s work involved in the project, using the AMSTAR2 Critical Appraisal tool for systematic reviews. Based on the findings, there is preliminary evidence for the potential of DBT to reduce self-harm behaviours and depression, although the evidence is low quality. Further research is necessary to increase our confidence in the effectiveness of DBT in the treatment of BPD.

## Background

Borderline personality disorder (BPD) is primarily a disorder of dysregulation of all aspects of emotional responding (Crowell et al 2009) with a persistent pattern of instability regarding self-image, interpersonal relationships, and impulsivity (International Classification of Diseases (ICD) 2021). The updated version of the ICD-11 defines personality disorder as a single condition with different severity levels without considering BPD as a separate condition (Bach et al 2022). The estimated prevalence of BPD globally in the general population is 1.6% and 20-25% among inpatients with psychiatric disorders (Ellison et al 2018).

BPD can have a considerable impact on one’s personal, social and occupational functioning (World Health Organisation, 2018). Finding it challenging to control their emotions and impulses (Storebø et al., 2020), people with BPD are at increased risk of depression, substance misuse, eating problems, and engaging in suicidal and self-harming behaviours (Kienast et al 2014, Paris 2019, Soloff et al 2000). Analysis of routine data has estimated the total service costs of care combined with costs of lost employment for people with a personality disorder in England to be £7.9 billion in 2007, with a projection of £12.3 billion by 2026 (McCrone et al 2008).

While a variety of psychotherapeutic approaches has been used in the management of BPD, Dialectical Behaviour Therapy (DBT) was designed specifically to treat deliberate self-harm (with or without suicide intent) in women with BPD (Linehan 1993; Samari et al., 2020). It has received the most research attention (Stoffers et al 2012) and is recommended as a treatment option for women with BPD to reduce self-harm in the UK (National Institute for Health and Care Excellence; NICE, 2009; updated in 2018), in the United States (American Psychiatric Association Guideline, 2001), and in Australia (National Health and Medical Research Council, 2012). DBT consists of four aspects: individual psychotherapy, telephone coaching, group skills training (ST), and therapist consultation teams (Linehan and Wilks 2015). It aims to teach clients behavioural skills that target common symptoms of BPD, including emotional lability and impulsivity such as self-injurious behaviours (May et al 2016). Standard DBT and DBT ST are commonly used approaches in the treatment of BPD and other mental health problems with similar aspects, such as issues with emotional regulation (Valentine et al., 2015).

While previous reviews (Kliem et al 2010, Oud et al 2018) have investigated the effects of DBT on impulsivity leading to self-injurious behaviours (self-harm, suicide), emotional aspects received less attention. Therefore, a review by Chen et al (2021) aimed to examine the effects of DBT on the self-injurious behaviours of self-harm and suicidal ideation, in addition to some of the emotional aspects of BPD, namely anger and depression, to provide a fuller picture of how DBT may help those with BPD.

### Aims of the commentary

This commentary aims to critically appraise the methods applied in a meta-analysis conducted by Chen et al (2021) examining the effects of DBT on the emotional aspects of BPD, in addition to self-harming behaviours and suicidal ideation, and to expand upon the findings in the context of clinical practice and future research.

### Commentary approach

This critical review utilises a RaCES (Rapid Conversion of Evidence Summaries) project methodology. RaCES projects are part of the Applied Research Collaboration Northwest Coast (ARC NWC) infrastructure funded by the National Institute of Health and Care research (NIHR) (https://arc-nwc.nihr.ac.uk/wp-content/uploads/2024/07/RaCES-what-we-do.pdf). The commentary is a collaborative work between academics, health and care professionals, and people with lived experience of the ARC NWC. RaCES projects critically appraise and rapidly convert systematic reviews into evidence summaries with the aim to build research capacity in health and care professionals, develop professional networks, and inform practice using the latest scientific evidence. In this project, the team worked with a mental health nurse who works with individuals who self-harm using a DBT approach and was interested in the evidence base behind this practice. The nurse selected a systematic review that was relevant for practice. Together, we examined the methods and results of the review and evaluated its quality by using the AMSTAR 2 Critical Appraisal Tool for systematic reviews (Shea et al 2017). The AMSTAR2 is a comprehensive instrument that enables the evaluation of systematic reviews that include randomised and/or non-randomised studies of healthcare interventions and assists decision makers in identifying high quality systematic reviews using real world observational evidence. The evaluation was performed by two researchers (KUG, JEH) and decisions were reached through discussion. The evidence was then summarised and based on its quality, explored in relation to practice and policy. Finally, a commentary piece was written up.

## Methods of the review by Chen et al (2021)

This is a protocol registered systematic review that searched multiple databases from date of inception to November 30, 2020, including hand searches of related studies. Only randomised controlled trials (RCTs) were included that compared various types of DBT (including standard DBT and DBT ST) with a control group of treatment as usual or non-dialectical therapy for patients with a BPD diagnosis aiming to reduce self-harming behaviours, suicidal ideation and/or emotional aspects (anger, depression). Appropriate data screening and data extraction was performed independently by two researchers and authors of included studies were contacted for any missing information.

While risk of bias was performed by two researchers independently, the bias assessment tool was not named in the report. Based on the reference used, the domains reported, and information from the protocol, the study appears to have used version 1 of the Risk of Bias tool for Randomised Trials (RoB; Higgins and Green 2008). This older version of the tool comprises of seven domains of bias. However, the authors carried out the assessment considering only six domains and determined the domain around blinding participants irrelevant. Based on the six domains, authors developed their own quality rating categories, A (low risk of bias), B (moderate risk of bias), and C (high risk of bias) with plans to exclude studies in category C with high risk of bias (disregarding the domain in blinding).

Meta-analyses were conducted to produce summary estimates using both random and fixed effects models to investigate the effects of DBT on self-harm, suicidal ideation, depression, and anger. The authors reported conducting several subgroup and sensitivity analyses to identify the cause of heterogeneity. Publication bias was assessed for studies with the outcome of ‘depression’.

## Results of the review by Chen et al (2021)

Eleven RCTs were included in the systematic review with a total of 849 participants investigating standard DBT (8 studies) or DBT ST (3 studies) in comparison to usual care (5 studies) and non-DBT (6 studies). Using their own categories, authors deemed two of the studies as low risk of bias (category A) and nine studies as moderate risk of bias (category B). Authors were primarily concerned about unclear reporting of allocation concealment and random sequence generation.

### Main analyses

Based on a meta-analysis of seven studies, DBT was found to have a statistically greater yet small effect in reducing self-harming behaviour in people with diagnosed BPD than usual care or other treatment (SMD=-0.28, 95%CI (-0.44, -0.12), p=.0005), with no heterogeneity detected among studies. Pooling the results from nine studies, the meta-analysis revealed a statistically significant small difference in reducing depression in the experimental group compared to the control condition (SMD = −0.34, 95% CI = (−0.53, −0.15), p = .0004). Moderate heterogeneity was found between groups, which was attributed to the study by Soler et al (2009) through sensitivity analysis, potentially due to the type and/or duration of the intervention. Meta-analysis of three studies investigating change in anger (SMD = −0.30, 95% CI (−0. 86, 0.27), p = .30) and suicidal ideation (SMD = −0.26, 95% CI (−0.74,0.21), p = .28) revealed no statistically significant differences between experimental and control groups. Heterogeneity was ‘substantial’ and ‘high’ for suicidal ideation and anger, respectively.

### Sub-group analyses

Sub-group analyses suggested that standard DBT, compared to DBT ST alone, had a more beneficial effect in reducing depression in the experimental group (SMD = −0.31, 95% CI (−0.51, −0.11), p = .0002). Additionally, according to a sub-group analysis to examine the effect of time, DBT statistically significantly reduced depression longer-term, but not within four months. Finally, an analysis of sub-groups of studies exploring their use of outcome measures concluded that studies that measured depression by the Beck Depression Inventory (Beck et al 1961) and the Hamilton Rating Scale for Depression (Hamilton 1960) were more likely to show a positive effect.

## Commentary

The AMSTAR2 Critical Appraisal tool for systematic reviews (Shea et al 2017) highlighted a number of limitations in the methodology of this review. The full completed AMSTAR2 checklist is available on request from the corresponding author. Out of 16 criteria, five were not met and five were only partially met. The population (e.g. age, gender) and comparator groups (e.g. intervention dose) were not described in detail, and information regarding intervention setting was not provided, which raises concerns around the indirectness of the evidence (Guyatt et al 2011). A major limitation of the study is disregarding the results in relation to blinding when quality assessing the studies. While it is challenging or, in many cases, may not be possible to apply blinding when delivering psychological interventions, the bias this introduces remains and needs considering. Indeed, a study by Juul et al (2020) found that only 3.2% of psychological studies reported blinding and only few considered the risk of bias this may have introduced, potentially overestimating the beneficial effects and underestimating the harmful effects of the interventions examined (Juul et al., 2020). Using all criteria to assess bias through the RoB determines that all included studies involved high risk of bias, which makes the findings of the individual studies, as well as the systematic review less conclusive. Furthermore, a new, revised version of the tool is available (RoB2; Sterne et al 2019), which was updated based on progress in research reflecting current understanding of how bias influences results. This may have been a more appropriate tool to use.

Publication bias was not found; however, due to the low number of studies, it was only assessed for studies with the outcome of depression, and therefore, it is unclear whether publication bias exists for the other outcomes included. In light of these methodological limitations and the high risk of bias involved in the primary studies included in this review, findings should be viewed with caution.

This review provides some evidence that DBT may reduce self-harm behaviours and depression, however, the quality of the evidence is very low based on GRADE criteria (Guyatt et al 2008). While the meta-analysis demonstrated statistically significant improvements for DBT, wide confidence intervals point to limited clinical significance. Only a limited number of studies was included that investigated the effects of DBT on anger and suicidal ideation and found no effect.

### Implications for practice and future research

In terms of psychotherapeutic interventions most of the evidence is on DBT with small to moderate effect (NICE 2009, updated in 2018) with regard to self-injurious behaviour and suicidality (Kliem et al 2010; Oud et al 2018), depression (Panos et al 2014), and anger (Ciesinski et al 2022). Additionally, DBT appears to be superior to other therapeutic modalities in terms of cost effectiveness (e.g. Brettschneider et al 2014, NICE 2009). While the evidence of the benefits of DBT for BPD is preliminary and of moderate quality (including both RCTs and non-RCTs; NICE 2009, updated in 2018), currently, it is the therapeutic approach with most empirical evidence. More robust RCTs are needed to provide support for its effectiveness with particular attention to sample size, heterogeneity of interventions and outcome, and attrition. Furthermore, it is key for future primary studies and systematic reviews to explore whom could benefit from DBT, how, in what setting, and under what circumstance.

## Conclusions

The empirical evidence on DBT in the treatment of BPD is limited, particularly with regard to the management of anger and suicidal ideation. Nevertheless, DBT is considered superior compared to other psychological approaches due to its larger evidence base and apparent cost-effectiveness (NICE, 2009, updated in 2018). However, as the evaluation of evidence in this commentary suggests, further research is necessary to increase our confidence in the effectiveness of DBT in the treatment of BPD.

## Relevance for clinical practice and further research

- There is some evidence that DBT may reduce self-harm behaviours and depression, however, evidence around its effects on anger and suicidal ideation is limited.
- It is unclear what population could benefit from DBT, how, in what setting, and under what circumstances. Further research is needed to identify these aspects.
- There is a need for more and better-quality research to investigate the effects of DBT on BPD, ideally in the form of randomised controlled trials, to provide support for its effectiveness.

## CPD reflective questions

- Why is DBT considered as a promising treatment approach for BPD?
- What are some of the methodological and other challenges when investigating the effectiveness of DBT for BPD?
- Think about aspects, such as setting, duration and dosage of therapy, and the population that receives this therapy. How may these affect intervention effectiveness?
